# Treatment of Sprains by “Firing”

**Published:** 1853-05

**Authors:** 


*Treatment of Sprains by "Firing."* By JAMES DICKINSON, Esq.

The advantages of "Firing" in many forms of rheumatic and neuralgic affections have been pointed out by Corrigan, Day, and others, (vide "Abstract," vol. III., p. 199.) Its use in sprains of the back, seems to be one from which the most striking benefit may be anticipated, as is seen in the following remarks:

Sprained backs are cases which give the surgeon much trouble and annoyance, appearing in many instances to resist every remedy. Many cases have come under my notice, and finding that blisters, cupping, stimulating liniments, &c., failed, I tried "firing," and the results have been most successful; patients who for many weeks have evinced the greatest agony, have, after the first or second application, been perfectly cured. The plan to be adopted is as follows: Heat a metal button, the shank of which is fixed into a wooden handle, to such a temperature as can be borne with slight pain. Pass it lightly over the affected part, without inducing vesication, which is unnecessary. The pain produced is severe, but is transient. In long-standing cases two or three applications are required; in recent ones one will be found sufficient.*—*Prov. Med. and Surg. Journal.*

* This remedy sometimes affords relief in cephalalgia, and other forms of neuralgia.—EDITOR BUFFALO MEDICAL JOURNAL.

